# Femtosecond Inscription of a Fiber Bragg Grating Spectral Array in the Same Spatial Location

**DOI:** 10.3390/s23084064

**Published:** 2023-04-18

**Authors:** Aviran Halstuch, Amiel A. Ishaaya

**Affiliations:** School of Electrical and Computer Engineering, Ben-Gurion University of the Negev, Beer-Sheva 8410501, Israel

**Keywords:** FBG fabrication, special fiber gratings, physical sensors

## Abstract

A five fiber Bragg grating (FBG) array is inscribed at the same spot with a single uniform phase-mask (PM). The inscription setup consists of a near-infrared femtosecond laser, a PM, a defocusing spherical lens and a cylindrical focusing lens. The tunability of the center Bragg wavelength is achieved by a defocusing lens, and by translating the PM, which results in a different magnification of the PM. A first FBG is inscribed, followed by four cascading FBGs, which are inscribed exactly at the same spot only after the translation of the PM. The transmission and reflection spectra of this array are measured, showing a second-order Bragg wavelength at ~1.56 µm with a transmission dip of ~−8 dB. The spectral wavelength shift between each consecutive FBG is ~2.9 nm, and the total wavelength shift is ~11.7 nm. The reflection spectrum of the third-order Bragg wavelength is measured at ~1.04 µm, showing a wavelength separation of ~1.97 nm between neighboring FBGs, and the total spectral span between the first FBG and the last one is ~8 nm. Finally, the wavelength sensitivity to strain and temperature is measured.

## 1. Introduction

Femtosecond-induced refractive index change in transparent materials was demonstrated for the first time twenty-five years ago [1]. Since then, this topic has been extensively researched, and many devices and applications have been demonstrated, such as waveguides, couplers, filters, and fiber Bragg gratings (FBGs), which were all obtained using femtosecond inscriptions [2]. For the last twenty years, femtosecond inscription of FBGs has been widely explored due to its crucial importance in the fields of optical fiber sensing and fiber lasers. Grating inscriptions in different types of optical fibers and with various focusing conditions have been demonstrated. Today, femtosecond inscriptions of FBGs are carried out through two major different techniques. First, the direct writing methods, which include the point-by-point (PbP) [3], line-by-line (LbL) [4] and plane-by-plane (PlbPl) [5] techniques. The grating points or planes are formed one-by-one in these techniques, which require very accurate air bearing stages, high-quality and high-numerical aperture objectives for focusing the light onto the fiber, and high control of the moving stages. Although these techniques are quite flexible regarding the choice of the center Bragg wavelength and the grating profile, they are usually also quite slow, since each grating plane is inscribed individually, one after the other. In addition, immersion oil and ferrules are very often needed for the inscription. The second major technique is the phase-mask (PM) technique, where the whole grating length is inscribed at once with a PM and a cylindrical focusing lens [6,7]. This technique is usually simpler than the direct writing techniques, more robust, and very often more suitable for different applications. However, the PM technique is less flexible, since the center Bragg wavelength is pre-determined by the PM grating period. The Bragg wavelength of the mth order is defined by [8]:

(1) $$m\cdot \lambda_{B,m}=2\cdot n_{eff}\cdot \Lambda_{FBG},$$

where λ_B_ is the center Bragg wavelength, n_eff_ is the effective refractive index of the fiber, Λ_FBG_ is the grating period in the fiber core, which is equal to half the PM grating period, and m is the grating order.

Fortunately, tuning the Bragg wavelength with the PM technique has been demonstrated by introducing controlled defocusing and aberrations to the inscription beam [9,10,11], stretching the PM [12], and using a spatial light modulator (SLM) [13], or a deformable mirror [14]. All these techniques modify the PM grating period, and, in turn, modify the grating period of the fiber Λ_FBG_, which results in tuning the Bragg wavelength, λ_B_. Another way to achieve tunability is to stretch the fiber prior to inscription, which results in shorter grating periods after the fiber strain is removed and once the inscription process is completed [15]. This technique, unlike the previous ones, only modifies the grating period of the fiber, Λ_FBG_, (the PM period is not changed). This results in tuning the Bragg wavelength, λ_B_, which is useful only for achieving blue shifts. Femtosecond inscription of phase-shifted gratings has been demonstrated using this technique [16]. Finally, with pre-and post-femtosecond exposure treatments, the Bragg wavelength, λ_B_, can be tuned by modifying the effective refractive index, n_eff_ [17,18,19]. In this case, small changes in both blue and red shifts can be achieved.

Today, fiber optical sensors based on FBGs are widely used in medical healthcare, environmental and biochemical applications, civil infrastructure, the petroleum industry, and in mechanical engineering [20,21,22,23]. In addition, fiber Bragg gratings are also used for filtering, and in optical fiber lasers for both output couplers and high-reflectance mirrors [24]. The major two application windows of FBGs are the telecommunication ~1550 nm wavelength-band (C-band), and the ~1060 nm wavelength-band, in which the gain spectrum of Ytterbium is located.

In this paper, an array of five FBGs is inscribed at the same spot using a single uniform PM. Each FBG is separated ~2.9 nm from its neighbor so that a total span of ~12 nm is reached. For achieving the wavelength tunability, a defocusing lens inserted into the optical inscription path in front of the PM is used. By changing the distance of the PM from the fiber core, the magnification of the PM grating period is changed; therefore, the grating period inscribed in the fiber core is also changed. All FBGs are inscribed at the same spot without moving the fiber. The second order Bragg wavelength of ~1.56 µm and the third Bragg order wavelength of ~1.04 µm are clearly visible. To the best of our knowledge, this is the first time an array of FBGs has been inscribed exactly at the same spot with such a wavelength separation. Moreover, only one uniform PM is used for the whole process. In the past, only two FBGs with a slight wavelength shift were inscribed at the same spot to achieve phase-shifted gratings [11,16,25,26]. In addition, such a grating array of five FBGs can be used to simultaneously measure temperature and or strain of a specific spot with different wavelengths.

**Figure 1:**
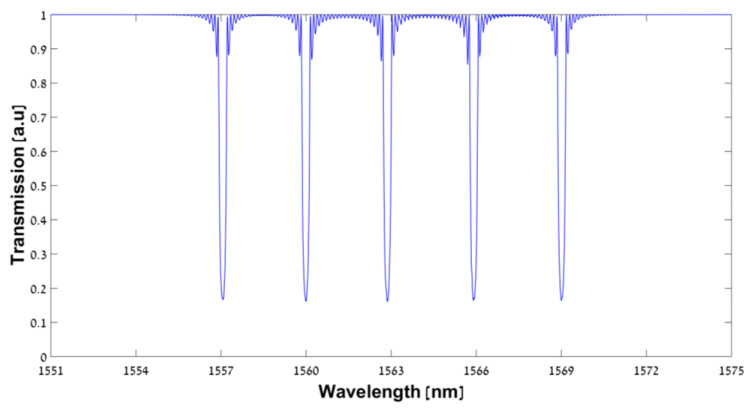
Calculated transmission spectrum of the complex structure, composed of five FBGs at different Bragg wavelengths, one on top of the other. The first Bragg grating wavelength is 1557 nm, followed by four others with a 3 nm separation between each two consecutive gratings. The transmission dip of each grating is ~80%.

To perform a simple estimation of the transmission spectrum of such a complex array structure consisting of five FBGs on the same spot, the thin film approach is employed [27]. The structure is described as five first-order gratings, with a 50% duty cycle. The total refractive index contrast is 0.0015, which is equally divided between all five gratings. The first grating is at a Bragg wavelength of 1557 nm, followed by the second one at a Bragg wavelength of 1560 nm, a third Bragg wavelength of 1563 nm, the fourth grating at 1566 nm, and finally, the fifth and last grating at 1569 nm. The transmission of this complex structure is solved, and the overall transmission is shown in Figure 1. The results show an array of five gratings with a wavelength separation of 3 nm between each two consecutive gratings, with each grating having a transmission dip of ~80%.

## 2. Experimental Setup Description

**Figure 2:**
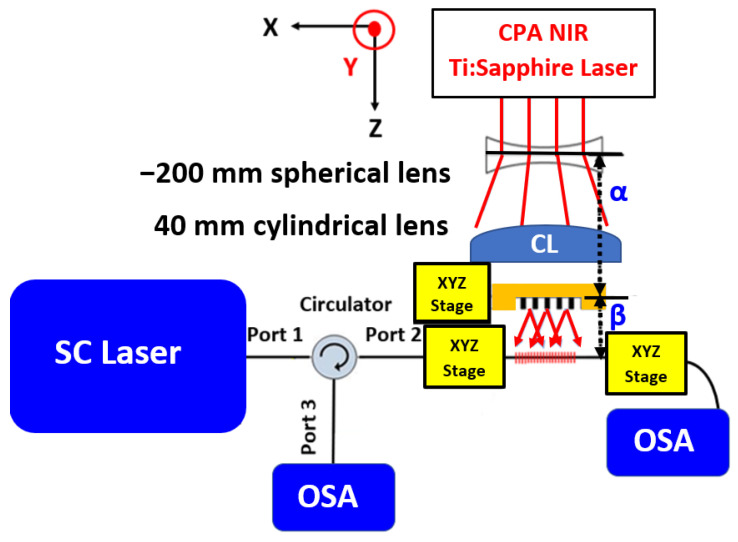
Experimental setup: A negative defocusing spherical lens with a focal length of f = −200 mm is positioned at a distance α in front of the PM. The PM is mounted onto an XYZ stage, which is located at a distance β in front of the fiber. A positive f = 40 mm cylindrical lens (CL) focuses the femtosecond inscription beam onto the fiber core. The fiber is connected to a broadband super continuum laser source (SC laser) via Port 1, and to an OSA at the other two ports via a circulator (Port 2 for transmission and Port 3 for reflection).

The configuration of the experimental setup is shown schematically in Figure 2. A commercial near-IR chirp pulse amplification (CPA) titanium–sapphire femtosecond laser system is used (Coherent Legend Elite). The CPA laser system produces 3.5 mJ per pulse at a 1 kHz repetition rate, while the center wavelength is 805 nm. The pulse duration, which is nearly transform-limited, is ~35 fs. The collimated Gaussian beam, which has a beam diameter of ~8 mm, is focused by a cylindrical lens through a PM on the fiber core into a line. The focal length of the cylindrical lens is 40 mm. The polarization of the CPA laser system is linear horizontal, (i.e., the electrical field oscillates in the X axis, which is parallel to the plane of Figure 2). The pulse energy of the inscription beam is controlled by rotating an achromatic half wave plate, which is placed in front of a stationary polarizing beam splitter. The outcome polarization from the beam splitter is perpendicular. Therefore, the polarization of the inscription beam relative to the PM is TE (i.e., the electrical field oscillates in the Y axis, which is perpendicular to the plane of Figure 2). The average power of the inscription beam is measured with an optical power meter (Coherent LM-10 HDT). The Ibsen Ltd. PM has a grating period of 2140 nm, which corresponds to a second-order Bragg grating at the telecommunication wavelength of ~1548 nm in a commercial single mode fiber (SMF). Less than 3% of the energy goes to the zero-diffraction order, while nearly 40% of the energy diffracts to each one of the first diffraction orders (the ± 1 diffraction orders). The remaining energy diffracts to higher orders and scatters. The PM is mounted onto a three-axis XYZ motion stage and is positioned ~4.4 mm in front of the fiber core to ensure a pure two-beam interference [28]. The fiber is held in two grooves, each on a three-axis XYZ motion stage and under controlled tension, which provides the high robustness of the inscription system. The Bragg grating size in the fiber core is calculated to be ~5 μm × 5 mm. This covers only part of the mode field diameter, which is ~9 μm for an SMF at the telecommunication wavelength of ~1.55 μm. Therefore, the focused inscription beam is scanned ± 10 μm around the fiber core (along the Y axis), perpendicularly to the fiber axis (the X axis), and to the laser inscription beam axis (the Z axis). Such scanning ensures proper coverage of the fiber core. In the other dimension, the Z axis, the depth of focus is much larger than the fiber core due to the extent of the Rayleigh length, which is ~75 μm. Therefore, there is no need to scan or move the writing inscription beam in the focusing axis (the Z axis) during the FBG inscription. Prior to the inscription, the commercial fiber (Corning SMF-28) is stripped from its 250 µm polymer coating. Then, the fiber is cleaned with ethanol and is placed onto the groves in the inscription setup. The fiber is connected through a circulator to a broadband supercontinuum laser source (YSL photonics Ltd., Wuhan City, China, Super Continuum Laser SC-5-FC) at one end (port 1), and to an optical spectrum analyzer (Yokogawa AQ6370D OSA) at the other two ends: port 2 for transmission and port 3 for reflection. During the inscription, the FBG is monitored with a spectral resolution of 20 pm and a sampling interval of 4 pm. In addition, a negative defocusing spherical lens with a focal length of f = −200 mm is added to the inscription beam at 163 mm in front of the fiber, and it remains stationary. This defocusing lens introduces a red shift to the Bragg wavelength due to PM magnification [9,11]. The center Bragg wavelength with a defocusing lens for the mth order is given by the following equation [11,29]:

(2) $$\lambda_{B,m}=n_{eff}\cdot \frac{f-\alpha -\beta }{f-\alpha }\cdot \frac{\Lambda_{PM}}{m},$$

where n_eff_ is the effective refractive index of the fiber, f is the focal length of the defocusing lens, α is the distance between the defocusing spherical lens and the PM, β is the distance between the PM and the fiber, Λ_PM_ is the PM period, and m is the Bragg order. Moving the defocusing lens towards the PM or away from the PM results in a different magnification of the PM grating period and will result in a red shift or a blue shift, respectively. For the stationary position of the defocusing spherical lens, the exact wavelength shift is determined by the PM position according to Equation (2), and by controlling the PM position (the distance to the fiber), the Bragg wavelength is tuned. The distance α + β is equal to 163 mm throughout the experiment, i.e., the position of the defocusing lens remains constant. However, by changing the distance β between the PM and the fiber core, the magnification of the PM and the Bragg wavelength is changed according to Equation (2). Moving the PM towards the fiber core increases α and decreases β. This results in a blue shift. Meanwhile, moving the PM away from the fiber core increases β, decreases α, and results in a red shift. This has been shown in previous works [11,29].

## 3. Femtosecond Inscription of an Array of Five FBGs

**Figure 3:**
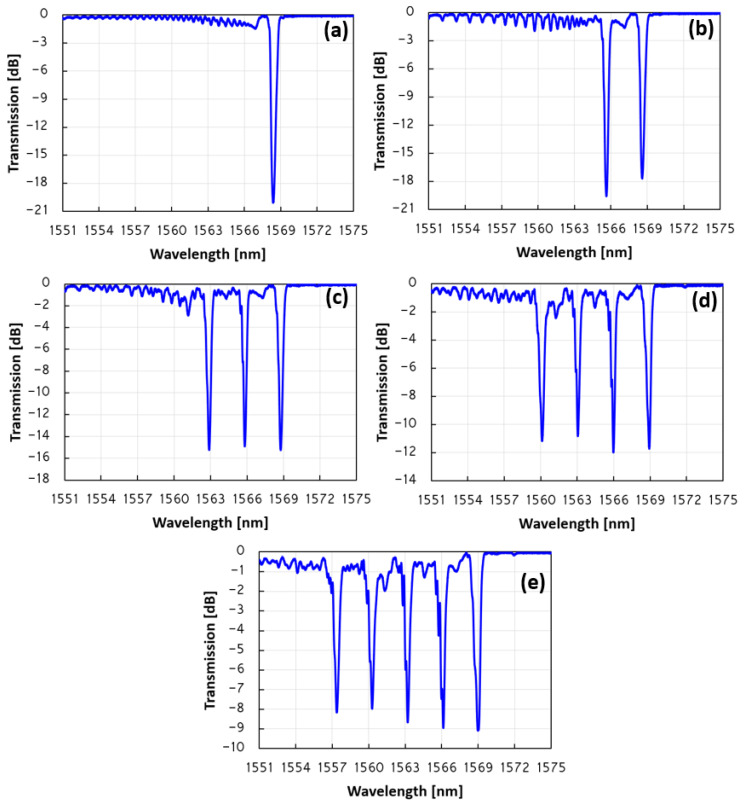
Measured transmission spectra of the FBG array. (**a**) First FBG inscribed with a pulse energy of 300 μJ for 90 s. The transmission dip is −20 dB, with a center Bragg wavelength of 1568.38 nm. (**b**) The measured transmission spectra of the two FBGs after the second FBG are inscribed with a pulse energy of 330 μJ for 90 s. The transmission dip of the second FBG is −19.5 dB, with a center Bragg wavelength of 1565.64 nm. Now, the transmission dip of the first FBG is −18 dB, with a center Bragg wavelength of 1568.58 nm (a 0.2 nm of red shift versus Figure 3a). (**c**) The measured transmission spectra of the three FBGs after the third one is inscribed with a pulse energy of 360 μJ for 90 s. The transmission dip of all three FBGs is −15 dB, with a center Bragg wavelength of 1562.88 nm, 1565.81 nm, and 1568.78 nm. The first two FBGs are red-shifted ~0.2 nm compared to the case shown in Figure 3b. (**d**) The measured transmission spectra of the four FBG array after the fourth FBG is inscribed with a pulse energy of 390 μJ for 90 s. The center Bragg wavelength of the array is 1568.93 nm, 1565.98 nm, 1563.04 nm, and 1560.1 nm. The transmission dip of the first two FBGs is −12 dB, and it is −11 dB for the other two. The first three FBGs are red-shifted compared to the case of Figure 3c. (**e**) The measured transmission spectra of the final five FBG array. The transmission dip of all five FBGs is clearly visible at approximately −8 dB, with a center Bragg wavelength of 1569 nm, 1567.15 nm, 1563.21 nm, 1560.29 nm 1557.39 nm. The ~2.9 nm separation between each two consecutive FBGs is clearly visible, as well as the 11.7 nm separation between the first FBG and the last one.

**Figure 4:**
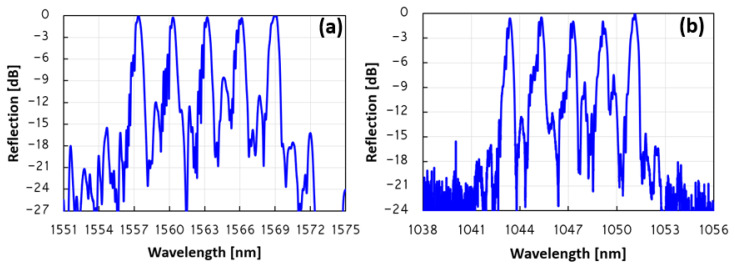
Measured reflection spectra of the final five FBG array. (**a**) The five transmission peaks are clearly visible at a wavelength of 1569.07 nm, 1566.17 nm, 1563.22 nm, 1560.29 nm 1557.37 nm. The ~2.9 nm separation between each two consecutive neighboring FBGs is clearly visible as well as the 11.7 nm separation between the first FBG and the last one. (**b**) The measured reflection spectra of the third-order Bragg of the five FBG array. The center Bragg wavelength of the five peaks and the ~1.9 nm separation between each two consecutive FBGs is clearly visible. All five peaks are approximately 20 dB above the background.

**Figure 5:**
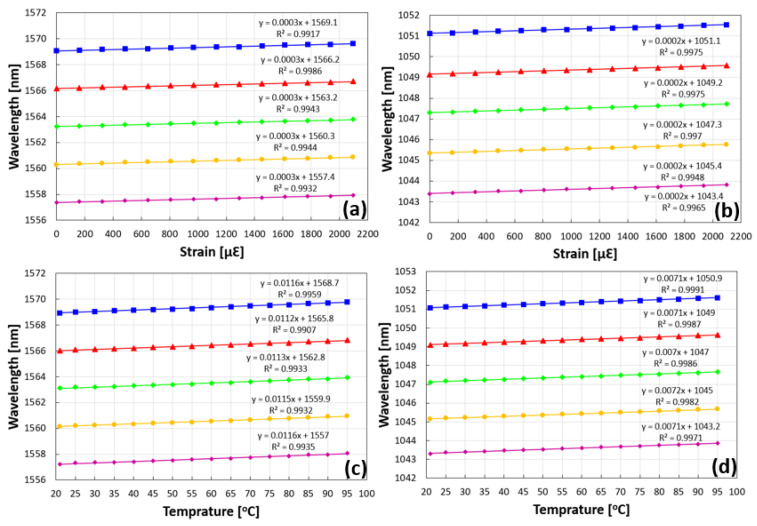
The measured wavelength sensitivity to strain (**a**,**b**), and temperature (**c**,**d**) for the five FBG array. (**a**) All five peaks show a strong linear dependence on strain with a similar sensitivity of 0.3 pm/µε. The R^2^ of the linear response is greater than 0.99 for all five peaks. (**b**) The measured wavelength sensitivity response to strain for the third-order Bragg of the five FBG array. All five peaks show a strong linear dependence on strain with a similar sensitivity of 0.2 pm/µε. The R^2^ of the linear response is greater than 0.99 for all five peaks. (**c**) The measured wavelength sensitivity of the second-order Bragg to temperature changes for the five FBG array. All five peaks show a strong linear dependence on temperature, with a similar sensitivity of ~11.5 pm/°C. (**d**) The measured wavelength sensitivity of the third-order Bragg to temperature changes for the five FBG array. All five peaks show a strong linear dependence on temperature, with a similar sensitivity of 7.1 pm/°C.

A five FBG array with a spectral separation of ~2.9 nm between each consecutive FBG is inscribed at a wavelength of ~1560 nm situated in the telecommunication wavelength band. The inscription procedure of the FBG array consists of the following steps. Once the polymer coating is removed and the fiber is attached to the inscription setup, an FBG grating is inscribed in the fiber core. The first FBG is inscribed for 90 s with a pulse energy of 300 µJ. The result is an FBG with a transmission dip of −20 dB and a center Bragg wavelength of 1568.38 nm, as can be seen in Figure 3a. Then, the femtosecond inscription beam is blocked, and the PM is translated 0.635 mm towards the fiber by increasing α by 0.635 mm and decreasing β by 0.635 mm, while α+ β remain the same (163 mm). The second inscription is made at the same spot without moving the fiber. According to Equation (2) for a second-order Bragg, a −200 mm spherical lens and a PM drift of 0.635 mm towards the fiber core should result in a blue-shift of 2.8 nm. After the PM is translated 0.635 mm towards the fiber, a second FBG is inscribed exactly at the same spot. The second FBG is inscribed for 90 s with a higher pulse energy of 330 µJ. A higher pulse energy is needed since the refractive index is already modified at this inscription position area; thus, the fiber is now more immune to refractive index modifications [17,18]. The result of this second inscription is a second FBG with a transmission dip of −19.5 dB at a center Bragg wavelength of 1565.64 nm, as can be seen in Figure 3b. In addition, the first FBG is now red shifted 0.2 nm from 1568.38 nm to 1568.58 nm and is also slightly reduced from −20 dB to −18 dB, as can be seen in Figure 4. The refractive index changes in the fiber due to the inscription are described by [8]:

(3) $$\Delta n=\Delta n_{DC}+\Delta n_{AC}\cdot \cos\left(\frac{2\pi }{\Lambda_{FBG}}z\right),$$

where ${∆n}_{DC}$ is the “DC” index change spatially averaged over a grating period, ${∆n}_{AC}$ is the refractive index change modulation and Λ_FBG_ is the grating period. There is an increase in the DC refractive index modification during the second inscription and now the AC refractive index modification increases for a different grating period (Λ_FBG_ is different for the second inscription). The result is an increase in the effective refractive index together with a reduction in the refractive index modulation (the AC refractive index change) of the first Bragg wavelength. This explains the red shift of the first FBG as well as the decrease in the transmission dip. These two phenomena have already been observed in a previous work [18]. When a post-femtosecond treatment (additional exposure) is carried out on a previously inscribed FBG, there is an increase in the DC refractive index, causing a red shift and a decrease in the AC refractive index (refractive index modulation), which causes a decrease in the transmission dip. Here, the second FBG is a “post femtosecond radiation treatment” for the first FBG. The third FBG is inscribed exactly at the same spot as the two previous ones, only afterwards, the femtosecond inscription beam is blocked, and the PM is again translated another 0.635 mm towards the fiber and away from the defocusing lens. The pulse energy for the third FBG is increased to 360 µJ, and the third FBG is inscribed for 90 s. The third FBG has a transmission dip of −15 dB and a center Bragg wavelength of 1562.88 nm, as can be seen in Figure 3c. In addition, now, both the first and the second FBGs are red shifted ~0.2 nm from 1568.58 nm and 1565.64 nm to 1565.81 nm and 1568.78 nm, respectively, as shown in Figure 5; this is the result of an increase in the DC refractive index. Moreover, there is another decrease in the transmission dip of both the first and second FBGs to −15 dB, which is the result of a decrease in the AC refractive index modification (refractive index modulation). The fourth FBG is inscribed in a similar manner. The femtosecond beam is blocked, and the PM is translated another 0.635 mm towards the fiber and away from the defocusing lens. Then, the inscription beam energy is increased to 390 µJ, and the beam is opened for 90 s. The result is another FBG with a center Bragg wavelength of 1560.1 nm and a transmission dip of −11 dB. Now, all three previous FBGs are red shifted ~0.2 nm from 1568.78 nm, 1565.81 nm, and 1562.88 nm to 1568.93 nm, 1565.98 nm, and 1563.04 nm, respectively, as can be seen in Figure 3d. In addition, the transmission dips of all three FBGs are reduced from −15 dB to −12 dB and −11 dB, as can be seen in Figure 3e.

This final inscription of 90 s is carried out after the PM is translated another 0.635 mm towards the fiber away from the defocusing lens, and the pulse energy is increased to 420 µJ. The result is a fifth FBG, with a transmission dip of −8 dB and a center Bragg wavelength of 1557.39 nm. All four previously inscribed FBGs are now red-shifted and their transmission dip decreases. The transmission spectra of the final FBG array are shown in Figure 3e. The center Bragg wavelength of the five FBGs array is 1569 nm, 1567.15 nm, 1563.21 nm, 1560.29 nm 1557.39 nm, and the transmission dip of this array is −9 dB, −9 dB, −8.5 dB, −8 dB, and −8 dB, respectively. Each FBG is separated ~2.9 nm from its neighbor, and the total span between the first FBG of the array and the last one is ~11.7 nm.

The reflection spectra of this FBG array can be seen in Figure 4a. The reflection peak of all five FBGs is quite similar and very close to 0 dB, while all five peaks are approximately 25 dB above the background. The measured bandwidth at −3 dB (FWHM) of each FBG in the array is ~0.55 nm, apart from the first inscribed FBG, which has a bandwidth of ~0.7 nm (the 1569 nm FBG). The measured insertion loss for the FBG array is approximately −0.5 dB. In addition, the third-order Bragg wavelength is also measured. The reflection spectra of the array of all five FBGs are clearly visible in Figure 4b. All five peaks are ~20 dB above the background. The reflection peaks are in the following wavelengths: 1051.12 nm, 1049.14 nm, 1047.16 nm, 1045.33 nm, and 1043.38 nm. This indicates a wavelength separation of ~1.9 nm between each two consecutive FBGs, in contrast to a wavelength separation of ~2.9 nm of the second-order FBG array. The total wavelength separation of the array between the first FBG and the last one is 7.74 nm.

## 4. Strain and Temperature Sensitivity

The FBG array wavelength sensitivity to strain and temperature changes for both the second- and third-order Bragg are measured. When the fiber is stretched axially, the grating period of the FBG increases, and the elasto-optic effect changes the effective refractive index of the fiber core. This results in a red shift when the fiber is stretched. For the strain sensitivity measurements, the five FBG array is fixed onto two stages. One side of the fiber is on a stationary stage, and the other is on a moving stage, while the FBG array is in the middle. The fiber is stretched with constant increments, while the wavelength of all five FBGs is monitored with the OSA. The initial distance between the two fixed points of the optical fiber is 70 mm. The fiber is stretched along the axis with increasing 10 µm increments each time. The measured sensitivity of the wavelength to strain changes for all five reflection peaks is found to be ~0.3 pm/με with excellent linearity. The linear fitting line of the wavelength shift of all five FBG peaks shown in Figure 5a indicates that the wavelength shift presents a high linear response to strain, with an R^2^ greater than 0.99 for all five peaks. The same experimental measurement is also made for the third-order Bragg wavelength. In this case, the measured wavelength sensitivity to strain for all five reflection peaks is found to be only ~0.2 pm/με, with excellent linearity. Such a sensitivity result is expected, since the wavelength is reduced by a third from ~1.56 µm to ~1.04 µm; thus, the sensitivity is also reduced by a third from ~0.3 pm/με for the second order to ~0.2 pm/με for the third order. The linear fitting line of the wavelength shift for the experimental results of all five FBG peaks of the third Bragg order is shown in Figure 5b. As in the case of the second order, here, the wavelength shift also has a high linear response to strain, with an R^2^ greater than 0.99 for all five peaks. While increasing the temperature of the fiber (FBG array), the thermal expansion of the fiber changes the period (Λ); this acts to increase the grating period, and the thermo-optic effect changes the effective refractive index (n_eff_). This results in an expansion of the grating period, and an increase in the effective refractive index of the fiber as the temperature of the fiber increases. Here, the temperature of the fiber (FBG array) is increased, and the wavelength shift is recorded with the OSA. The result is a red shift of the central second-order Bragg wavelength, as can be seen in Figure 5c. From the linear fitting of the experimental points, one can find that the wavelength sensitivity to temperature changes is ~11.5 pm/°C for all five peaks of the FBG array. The linear fitting line of the wavelength shift is shown in Figure 5c. Here, the wavelength shift presents a high linear response to temperature changes, with an R^2^ greater than 0.99 for all five peaks of the grating array. The same measurement is also taken for the third-order Bragg. The temperature of the fiber is increased, and the wavelength shift is monitored with the OSA. Again, the results in Figure 5d show an excellent linear response to temperature, with a sensitivity of ~7.1 pm/°C for all five peaks of the FBG array. The R^2^ of the wavelength sensitivity to temperature is greater than 0.995 for all five peaks. In this case, the smaller temperature sensitivity of ~7.1 pm/°C for the third-order Bragg, compared to the temperature sensitivity of ~11.5 pm/°C for the second-order Bragg, is also expected, since there is a reduction by one third in the wavelength from ~1.56 µm to ~1.04 µm.

## 5. Conclusions

To summarize, a very simple novel method for same spot inscription of a five FBG array with one single uniform PM is presented. This technique is based on the same spot inscription of cascaded FBGs, having a center Bragg wavelength shift from each other, while the inscription process is carried out by a femtosecond laser. The wavelength shift is achieved by an additional defocusing spherical lens which is placed in the inscription beam in front of the PM, thereby modifying the PM grating period. By translating the PM towards the fiber and away from the defocusing lens between each inscription, a different magnification of the PM period is achieved, resulting in a different center Bragg wavelength of each FBG. All five FBGs are inscribed at exactly the same spot. This method is experimentally demonstrated on a commercial Corning SMF-28 fiber at a wavelength of ~1.56 µm, achieving a total wavelength span of ~11.7 nm for the five FBG array, while each FBG is separated by ~2.9 nm from its neighbor. At the end of the inscription process, the transmission dip of each FBG is approximately –9 dB. During the inscription process of a new FBG, all previously inscribed FBGs are red-shifted, and their transmission dip decreases. The first FBG is reduced by more than 10 dB from –20 dB to –9 dB, and it is red-shifted by more than 0.6 nm. This phenomenon has also been observed on a single inscribed FBG, when post femtosecond photo treatment yielded a red-shift together with a decrease in the transmission dip caused by the increase in the effective refractive index (n_eff_) and a decrease in the refractive index modulation [18]. Finally, the wavelength sensitivity to strain and temperature changes is also measured at ~1.56 µm (second-order Bragg) and at ~1.04 µm (third-order Bragg). For the second-order Bragg, a sensitivity of ~0.3 pm/µε and a sensitivity of ~11.5 pm/°C are measured for strain and temperature changes, for all five peaks, respectively. For the third-order Bragg, the sensitivity of the array is reduced roughly by a third, as expected. In this case, the wavelength sensitivity to strain and temperature is ~0.2 pm/µε and ~7.1 pm/°C, respectively. Such a grating device can be used for measuring the temperature and strain of a specific point in the fiber simultaneously at different wavelengths. This device can be used also in fiber lasers, while lasing at different wavelengths simultaneously and measuring the temperature and/or strain at another wavelength. Future work may include investigation of the polarization sensitivity and inscription of six or more FBGs at other wavelengths and with larger spectral separations.

## Data Availability

The data underlying the results presented in this paper are not publicly available at this time but may be obtained from the authors upon reasonable request.
